# On the Role of LGN/V1 Spontaneous Activity as an Innate Learning Pattern for Visual Development

**DOI:** 10.3389/fphys.2021.695431

**Published:** 2021-10-27

**Authors:** Sahar Behpour, David J. Field, Mark V. Albert

**Affiliations:** ^1^Department of Information Science, University of North Texas, Denton, TX, United States; ^2^Department of Psychology, Cornell University, Ithaca, NY, United States; ^3^Department of Computer Science and Engineering, University of North Texas, Denton, TX, United States; ^4^Department of Biomedical Engineering, University of North Texas, Denton, TX, United States

**Keywords:** spontaneous activity, self-organization, efficient coding, innate learning, criticality

## Abstract

Correlated, spontaneous neural activity is known to play a necessary role in visual development, but the higher-order statistical structure of these coherent, amorphous patterns has only begun to emerge in the past decade. Several computational studies have demonstrated how this endogenous activity can be used to train a developing visual system. Models that generate spontaneous activity analogous to retinal waves have shown that these waves can serve as stimuli for efficient coding models of V1. This general strategy in development has one clear advantage: The same learning algorithm can be used both before and after eye-opening. This same insight can be applied to understanding LGN/V1 spontaneous activity. Although lateral geniculate nucleus (LGN) activity has been less discussed in the literature than retinal waves, here we argue that the waves found in the LGN have a number of properties that fill the role of a training pattern. We make the case that the role of “innate learning” with spontaneous activity is not only possible, but likely in later stages of visual development, and worth pursuing further using an efficient coding paradigm.

## What Is the Purpose of Spontaneous Activity?

Highly organized patterns of spontaneous activity are observed across the developing sensory systems of a wide variety of species. These activity patterns are robust to perturbations (Turrigiano, 1999; Blankenship and Feller, 2010). Several studies suggest the patterns themselves carry information that guides nervous system development in areas as diverse as the retina, cochlea, spinal cord, cerebellum, hippocampus, and neocortex (Blankenship and Feller, 2010; Dehorter et al., 2012) and altering patterns of activity leads to poorer network refinement (Huberman et al., 2008). Therefore, these correlated patterns are crucial for neural circuit maturation (Kirkby et al., 2013). A collection of past and current discoveries about spontaneous activity in both developing and mature sensory systems is provided by (Imaizumi et al., 2018). Although there remains debate regarding the precise role the activity plays, it is necessary to establish critical neural circuits before eye-opening (Shatz and Stryker, 1988; Wong and Ghosh, 2002; Uesaka et al., 2006; Kim et al., 2009).

One fascinating form of spontaneous activity is found in the *“retinal waves” of* the developing retina. Retina is a layer of tissue in the back of the eye of most vertebrates and some mollusks that senses light and sends images to the brain. Retinal waves are spontaneous bursts of action potentials that occur roughly once per minute and propagate in a wave-like fashion across the developing retina. They have been demonstrated in a majority of common vertebrates, including chicks (Sernagor et al., 2001), turtles (Evelyne Sernagor and Grzywacz, 1999), cats (Meister et al., 1991), rats (Galli and Maffei, 1988; Maffei and Galli-Resta, 1990), ferrets (Feller et al., 1996a), primates (Warland et al., 2006), and mice (Maccione et al., 2014). This spontaneous retinal wave phenomenon that occurs before the eyes first open was found and described in the developing mammalian over two decades ago; for complete references, see (Zhou, 2001).

Using a multielectrode array (Meister et al., 1991; Wong et al., 1993) or fluorescence imaging of calcium indicators (Wong et al., 1995; Feller et al., 1996b), the spontaneous activity in the developing retina has been monitored across large areas. The role of cholinergic circuits in driving this activity bursts of action potentials was indicated by recording the spontaneous bursts in the neural rabbit retina and blocked by nicotinic acetylcholine receptor analogies. About 10years later, Vivo recordings from fetal rat pups showed that neighboring ganglion cells fire periodic correlated action potential bursts that propagate like waves across the ganglion cell layer of ferret retinas, according to multielectrode array experiments. In addition, using calcium imaging sensitive dyes, videos of these endogenous patterns were recorded and demonstrated that correlated calcium transients propagate across the ganglion cell layer of the retina in a wave-like manner (Feller et al., 1996a).

While calcium imaging experiments of the intact retina showed the qualitative nature of the retinal waves, the characteristics of these waves, such as wave shape, speed, size, and duration using physiological details like dendritic field size and measured cell spacing, have been explored by neural models of various studies (Burgi and Grzywacz, 1994; Butts et al., 1999; Godfrey and Swindale, 2007). The spontaneous activity of the retinal waves is far from random, with more structure than one finds in simple models of correlated noise. They spread as amorphous bursting neural activity patterns with relatively slow dynamics. For instance, the average time between retinal waves in a given location is a median of 115s in the ferret retina. Typically, the patterns form well-bounded shapes that expand, distort, and dissipate over time. In the ferret retina, the average time between retinal waves in a given location is a median of 115s (Feller et al., 1996a); however, a ganglion cell’s burst of activity lasts roughly 1s, and the average wave propagation speed is 177μm/s (Feller et al., 1997). Therefore, the spontaneous activity in retinal waves is so robust that the possibility of noise contamination from instruments is not a significant concern.

Spontaneous activity patterns propagate through many parts of the developing nervous system to shape the wiring of emerging circuits. Prior to eye-opening, these activity waves originate in the retina and propagate to the superior colliculus (Ackman et al., 2012) and through the LGN of the thalamus, a multilayered structure that receives input from both eyes to build a representation of the contralateral visual hemifield, to the V1. LGN waves can correlate between the eyes. However, retinal waves cannot. Training based on the retinal waves produces a single eye vision, monocular model, versus LGN, which provides a binocular model. Three distinct higher ganglion cells, magno, parvo, and konio, compress and transfer the generated information by cone photoreceptors to processing centers in the retina. These cells, organized in different layers of LGN and the V1, have distinct functional and structural characteristics. In this paper, for the simplicity of the model in addressing critical elements for the disparity-selective vision, we do not distinguish between the three cellular subcortical pathways.

Spontaneous neural activity is correlated with brain functionality and its anatomical structures (Tozzi et al., 2016). This activity plays a critical role in the functional organization of the nervous system’s standard architecture (Cole et al., 2014), is generated by brain circuits, and provides a window into their dynamic operations (Omer et al., 2019). A number of computational models have shown how different forms of spontaneous activity can produce a topographic map of neural responses as found in the primary visual cortex (V1) [see (Erwin et al., 1995; Miikkulainen et al., 2006; Goodhill, 2018) for reviews]. Previous studies demonstrate that ongoing activity in V1 is not noise, though it may be a form of information processing (Arieli et al., 1995, 1996; Tsodyks et al., 1999; Kenet et al., 2003; Gutnisky et al., 2017). Following this, multiple studies performed on human and other animals discovered that this activity has a major role in shaping perception and behavior (Wagner et al., 1998; Ress and Heeger, 2003; Dehaene and Changeux, 2005).

## The Efficient Coding Paradigm

There is a wide variation in the nature of the spontaneous activity offered by different models. For example, von der Malsburg’s initial model (Malsburg, 1973) used simplistic bar-like stimuli, Linsker’s model (Linsker, 1986) begins with uncorrelated noise, Miller’s model (Miller, 1994) uses radially symmetric functions which represent the amount of correlation between units. Many such models rely on constraints in the neural connectivity (e.g., prespecified or adaptive lateral connectivity and dendritic field sizes) for receptive field formation with less emphasis on the precise nature of the activity. This can be contrasted with models of spontaneous neural activity that are primarily aimed at recreating the larger-scale statistical properties of the generated patterns (Butts et al., 1999; Godfrey and Swindale, 2007; Godfrey et al., 2009; Richter and Gjorgjieva, 2017). To find an excellent review of theoretical models of neural development, see (Goodhill, 2018). Here, we discuss this can be contrasted with computational approaches to map formation which use natural images as the input stimuli (Barrow et al., 1996; Shouval et al., 1996; Weber, 2001; Hyvärinen et al., 2009), In these models, cortical maps form due to the statistical properties of presented natural images.

There is a subset of developmental models which use natural images in addition to spontaneous activity; they apply the same learning method both before and after visual experience (Bednar and Miikkulainen, 1998; Burger and Lang, 1999; Bednar, 2002). These V1 cortical models have produced 2D maps which vary characteristically with orientation, ocular dominance, direction selectivity, spatial frequency, or a combination of these dimensions. The primary goal of these models was to produce a 2D cortical map and receptive fields in a physiologically plausible way. As with many models, the necessary constraints can either be in the network implementation or the statistics of the input; many of these models lie along this continuum, and consequently, there is a wide variety of network implementations. One simplification to this modeling approach is to focus at the computational, rather than algorithmic, level (Marr, 2010) evaluate a simple coding objective of V1, rather than an intricate network implementation, on natural and spontaneous inputs. Although the previous neural modeling techniques may produce a similar code, the particular learning approaches are less clear than simple statements of statistical independence or sparse coding.

We argue that an efficient coding approach would focus efforts on the sufficient statistical properties of spontaneous activity and natural scenes and less about the particular details of implementation. In practice, efficient coding as a computational approach explains how the brain represents and interprets information from the outside world and maximizes a particular metric of efficiency (Barlow, 1961). The efficient coding hypothesis proposes that early sensory processing aims to reduce redundancy (Barlow, 1961; Field, 1987). Sparse coding and independent component analysis (ICA) objectives are driven from the efficient coding hypothesis, each with a specific statistical method. Sparse coding is a type of unsupervised learning method of over-complete bases for efficiently representing the data. Sparse coding aims to represent information with as few simultaneously active neurons as possible in a large population.

The input vector $x_{i}$ is defined as a linear combination of basis vectors, which is the set of basis vectors. Independent codes can also be produced using ICA (Comon, 1994). Mathematically, ICA creates components through linear combinations of features with maximally and statistically independent responses under a specific set of assumptions. In other words, ICA transforms the observed data as a random vector into a vector of maximally independent components S measured by some function $s_{1},s_{2}..,s_{n}$ using the following linear transformation;


S=Wx

where $S={\left(s_{1},s_{2}..,s_{n}\right)}^{T}$ is a hidden component, and $X={\left(x_{1},x_{2}..,x_{n}\right)}^{T}$ is the observed data representation.

Unsupervised learning methods, such as ICA, with the goal of maximizing statistical independence, are successful in creating meaningful components. ICA was originally developed to address the blind source separation problem (Jutten and Herault, 1991) and has been particularly useful for problems with linear mixings, such as the classic cocktail party problem (Cherry, 1953; Haykin and Chen, 2005; Bronkhorst, 2015).

This efficient coding strategy has been successfully applied to various modalities, natural/non-natural images, and sounds (Urs et al., 2021). A self-contained Jupyter notebook of the efficient coding strategy is provided for the ease of experiments in computational neuroscience and computer science. However, in this study, our objective is to show that using the same strategy on LGN/V1 spontaneous activity patterns, it is possible to derive filters of those images similar to the Gabor filters. Therefore, we can use the extracted filters for future training task-oriented deep learning models. We follow a five-step strategy to apply efficient coding principles to V1/LGN spontaneous activity;

1. Data Collection: Collecting samples of spontaneously generated activity pattern images corresponding to the simulation of active ganglion cells in the retinal model, LGN, and V1.
2. Patch Extraction: Randomly extracting patches of these images, display them, and then reshape them accordingly.
3. Efficient Encoding: Applying encoding algorithms, that is, ICA using our self-contained Jupyter notebook (Urs et al., 2021).
4. Displaying Efficient coding Filters: Reducing the process used in patch extraction and flattening to display the resulting filters found through ICA.
5. Comparing to Experimental Neural Receptive Fields: Drawing comparisons between experimentally recorded receptive fields and the resulting filters from spontaneous neural activity.

The efficient coding approach, using sparse coding/ICA, has been successfully applied to activity patterns resembling retinal waves (Albert et al., 2008). This particular study investigated whether this activity could serve as a training pattern and produce the same type of V1 filters that are produced from natural scenes. The spontaneous activity model used was an abstraction of previous, more physiological models (Butts et al., 1999; Godfrey and Swindale, 2007; Godfrey et al., 2009). The three parameters of the model were directly related to known parameters in retinal wave physiology (fraction of recruitable amacrine cells, dendritic field sizes, and threshold number of neighboring amacrine cells needed to fire). The results showed this abstract, physiological spontaneous activity is capable of forming V1 receptive fields in the same way as an efficient coding of natural scenes. This model of development is not only straightforward conceptually, but elegantly simple given a neural implementation. This fits well with the recommendation for modeling approaches to V1 development in the (Swindale, 1996) review, “Remove as much detail as possible from your model, without reducing its descriptive scope.” Such simple innate learning strategies could be used as a bridge between molecular guidance cues forming crude receptive fields and adaptation using visual input after eye-opening. Most importantly, it is one more way animals that require a functioning visual system early in life may be able to adequately prepare the visual code in time for use.

It has been shown that spontaneous activity generation and propagation correspond to the percolation networks used in applied math and statistical physics (Hesse and Gross, 2014). As a typical example, the percolation theory involves networks with t=1, where t is the number of active points within distance r, often r=1. When “p,” a random fraction of the points on the grid square array of points, approaches a value known as the percolation threshold, “pc,” the pattern of activity is known to be fractal – the image statistics appear similar at all scales (Li et al., 2021). What we discuss in this paper is a system, an abstraction of hypothesized LGN and V1 developmental activity patterns, that can generate fractal patterns. To generate such large waves, we are approaching the percolation threshold. The whole reason for getting patterns at such a large scale is that there are advantages to getting self-similar patterns. That way, we can have systems trained at multiple scales. The method used to generate the patterns is based on a convergence of two general models: One is a percolation network that resembled retinal waves both visually and in how the patterns are generated (e.g., amacrine cells spontaneously firing and being recruitable) and the additional element is simply communication across eye layers (due to feedback to the LGN). We consider this model the minimal approach to create a binocular spontaneous activity pattern capable of producing disparity selective cells while still resembling retinal wave generation. The model can be set such that those spontaneous activity patterns have disparity distributions like in natural visual experience – which would have a beneficial effect in early learning, as evidenced by the disparity profiles of filters created through efficient coding. Similarly, other properties of the produced filters should approximately match natural scene statistics. This concept constrains the statistical properties of LGN waves to be ones in which they produce neural receptive fields on efficient coding.

There are significant principle advancements between this current position paper and the previous study by (Albert et al., 2008). First, this study proposed the role of V1/LGN spontaneous activity patterns, whereas, in the previous study, the focus has only been on V1. Second, the main goal of the previous paper was to show that an efficient coding algorithm can be applied for both natural and spontaneous activity inputs; however, in this perspective, this efficient coding strategy is introduced as a practical tool to create efficient codes of LGN/V1 activity patterns for further training roles.

## Could the Measured Endogenous Activity Be a Potential Training Pattern For Lgn-V1 Neurons?

In the case of retinal waves, where the vast majority of experimentation on endogenous activity has focused, the extent of training is limited. One immediate objection is that the waves are not binocular. Days after birth, primates have an adult-like proportion of disparity selective cells (Chino et al., 1997) and retinal waves are not correlated between the eyes to promote such selectivity. Also, the retinal waves of the ON and OFF-center cells are more independent at later stages (Wong et al., 1993), but the responses of these cell classes are intimately related in V1 neurons. Despite the popularity of retinal spontaneous activity, such concerns have turned many researchers away from considering the possibility of an innate learning role for spontaneous activity.

Here, we draw attention to a form of spontaneous activity that does not suffer from the many deficiencies of retinal waves as a training pattern – activity originating in the LGN. Although much less is known about this activity, we believe that it may provide additional insights into the early development of neural properties in the visual cortex. The endogenous activity in the LGN significantly overlaps in time with V1 activation (Wong et al., 1993), thus being able to directly influence V1. The activity is also correlated between eye-specific layers, as well as ON and OFF-center cell layers (Weliky and Katz, 1999). Finally, the evidence from electrode arrays shows that the activity appears as a wavefront moving across these layers. By all accounts, LGN endogenous activity appears better suited as a potential source of innate training patterns.

There are potential confounds with such an assessment of LGN activity. First, it is more difficult to experimentally characterize LGN waves through electrode penetrations in comparison with the characterization of retinal waves using calcium imaging. Also, proper LGN activity requires V1 feedback; there is no correlated spontaneous activity between eye-specific layers in the LGN without feedback (Weliky and Katz, 1999). The alleged source of training patterns for V1 may more correctly be called dynamic LGN/V1 activity, making a label for the source of such activity more complicated than a simplistic, one-area label as in retinal waves. Ultimately, from a computational perspective, the specific source of the activity is not as critical as the statistical properties of the resulting activity. What is important for this paradigm is the existence of a functional separation between an innate training pattern (LGN/V1 waves) and efficient coding technique (e.g., sparse coding/ICA). These alternate roles need not be in physically separate locations. For example, this paradigm does not preclude the possibility of neural models with activity and adaptation occurring within V1 (Grabska-Barwinska and von der Malsburg, 2008), although here we discuss the combination of the LGN and V1 activity.

Our previous work pointed to this LGN/V1 activity as a potential source of visual training patterns. Endogenous activity is how the system could, in theory, bridge between molecular guidance cues and real-world experience for development. Beyond this, there are a number of parallels that can point toward a more complete interpretation of this activity. Activity model parameters have been directly related to similar parameters in physiological models. Receptive fields derived from these activity patterns are also qualitatively similar to experimental data. In addition to monocular and binocular properties of newly developed cells, the temporal component of this activity can be included in a more thorough analysis. A caveat to these models is that they are not meant to match adult coding performance – both in modeling and in animal development. For most animals, the goal in early visual development is to have a system that is not equivalent, but closer to the adult visual code than what can be produced by molecular guidance cues alone.

The nature of the explanation mentioned here is computational, although many other levels of understanding the role of this activity fit this framework (Freeman, 1989). On the level of implementation, endogenous activity is necessary for strengthening and guiding synaptic connections through synaptic pruning (Shatz and Stryker, 1988), axon branching (Hosokai et al., 2006), and dendritic patterning (Wong and Ghosh, 2002). This interpretation is not only compatible with a computational understanding, but a necessary part of a neural implementation. A number of neural models have implemented development of a topography and individual receptive fields. A subset of these neural models may be compatible with the computational, statistical interpretation presented here. The specific developmental algorithm may vary, but the goal of applying an efficient coding strategy (e.g., sparse coding/ICA) both before and after experience may be universal.

## What Is the Role of Innate Learning with Spontaneous Activity in the Visual System?

Efficient coding/computational approaches have added to our understanding of adult V1 by providing an additional functional role. Neural response of simple cells in V1 can be understood by their neural connections in cortex (implementation, “how”), their visual receptive fields and generalized neural models (algorithm, “what”), and also by the goal of the neural processing (computation/efficient coding, “why”). Previous work (Olshausen and Field, 1996; Bell and Sejnowski, 1997) has shown that the unique, Gabor-like receptive fields of V1 simple cells can result from an efficient coding of natural scenes. The underlying hypothesis, that V1 receptive fields can be understood as the result of a general computational strategy, has led to an explosion of techniques trying to both capture the statistical properties of natural scenes and relate those properties to V1 cell responses.

In the same way that efficient coding applied to natural scenes has led to a deeper understanding of the role of V1, similar computational strategies can lead to a deeper understanding of spontaneous activity. The same efficient coding strategy learning both on endogenous activity and external, natural stimuli may help explain many of the statistical properties of spontaneous activity deeper than retinal waves. This has the potential to build bridges between development and experience-based learning in areas that are often treated separately. Much of the criticism of this approach leveled at retinal waves does not hold when considering combined LGN/V1 activity, permitting further work in this domain to continue. Current efficient coding “innate learning” models show promise and point toward further computational, theoretical, and experimental work.

**Figure 1:**
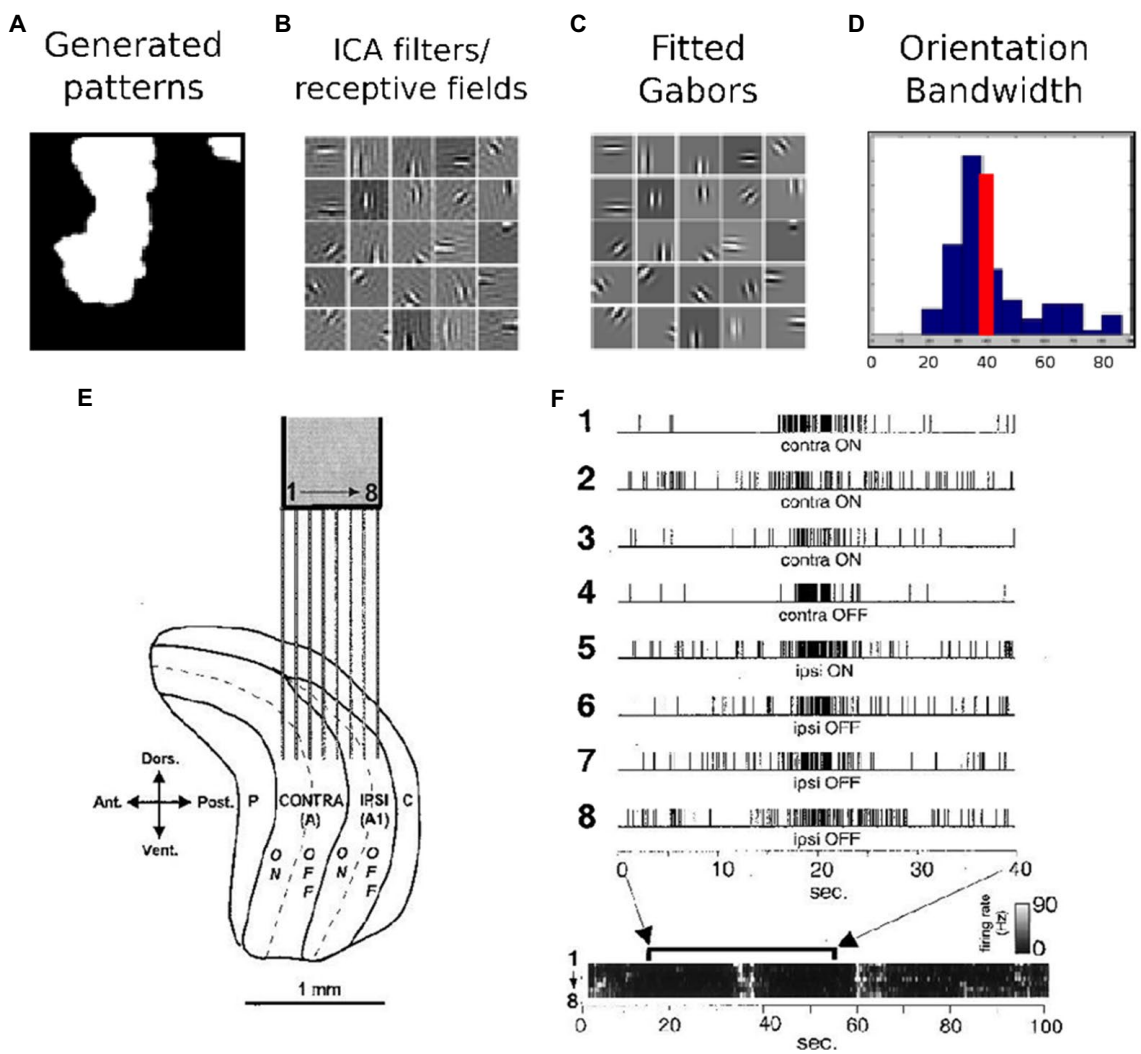
Early experimental and theoretical results suggesting the training role of LGN/V1 activity. **(A–D)** Efficient coding results demonstrating that a simple model of spontaneous activity can produce receptive fields with adult-like receptive field properties – details in (Albert et al., 2008). **(A)** An example spontaneous activity pattern, **(B)** the resulting receptive field after applying ICA, **(C)** Gabors fit to these receptive fields, **(D)** a histogram of orientation bandwidth with the red line indicating the average adult V1 primate orientation bandwidth for comparison. **(E,F)** Early experimental results indicating correlated LGN spontaneous activity across eye layers from (Weliky and Katz, 1999), reprinted with permission; electrodes 1–4 are in the contralateral eye layer, while −8 are in the ipsilateral eye layer.

The training role of LGN/V1 activity is demonstrated through early experiments and theoretical results, as is shown in Figure 1. According to (Albert et al., 2008), a simple model of spontaneous activity can produce receptive fields with adult-like receptive field properties. This has been done using the efficient coding strategy, mainly ICA applied on images of natural spontaneous activity patterns. In Figure 1A, an example of such a spontaneous activity pattern is given. After applying ICA on this set of data, the resulting filters and receptive fields are represented in Figure 1B. A visual comparison shows that Gabor filters, shown in Figure 1C, can properly fit these generated receptive fields by ICA. A histogram of orientation bandwidth, with a red line which indicates the average adult V1 primate orientation bandwidth, is used for the comparison and is shown in Figure 1D. Correlated LGN spontaneous activity across eye layers from (Weliky and Katz, 1999), reprinted with permission; electrodes 1–4 are in the contralateral eye layer while 5–8 are in the ipsilateral eye layer, indicated in Figure 1E,F. Ultimately, the goal is to answer the same question that is naturally posed when one is first exposed to the highly structured, spontaneous patterns, “What is the purpose of this activity?”

## Summary

Spontaneous neural activity plays a significant role in visual development. Although this activity in the retina is widely discussed, it is less investigated for LGN. In this study, we argue that LGN spontaneous activity may provide additional insights into the early development of neural properties in the visual cortex. We claim that the waves found in the LGN have several properties that fill a training pattern’s role. Thus, the role of “innate learning” with spontaneous activity in later stages of visual development is possible and worth pursuing further using an efficient coding paradigm.

## Data Availability Statement

The original contributions presented in the study are included in the article/supplementary material, and further inquiries can be directed to the corresponding author.

## Author Contributions

All authors listed have made a substantial, direct and intellectual contribution to the work, and approved it for publication.

## Conflict of Interest

The authors declare that the research was conducted in the absence of any commercial or financial relationships that could be construed as a potential conflict of interest.

## Publisher’s Note

All claims expressed in this article are solely those of the authors and do not necessarily represent those of their affiliated organizations, or those of the publisher, the editors and the reviewers. Any product that may be evaluated in this article, or claim that may be made by its manufacturer, is not guaranteed or endorsed by the publisher.
